# DIFFERENCES IN THE EXPERIENCES AND BEHAVIORS OF OLDER ADULTS IN NSHAP DURING THE COVID-19 PANDEMIC

**DOI:** 10.1093/geroni/igac059.116

**Published:** 2022-12-20

**Authors:** Louise Hawkley, Dawn Carr

## Abstract

This symposium uses data from the National Social Life, Health & Aging Project to examine how and for whom older adults’ social relationships and experiences changed with the advent of the COVID-19 pandemic. Zhang et al. examine video call usage among older adults with hearing impairment and how this might mitigate loneliness. They find that although this population reported greater loneliness during the pandemic, video calls attenuated this relationship in a dose-response manner. Wilder et al. evaluate 2015 resilience and socioeconomic status as predictors of older adults’ reports that the pandemic led to a positive change in their lives. Results revealed greater odds of reporting a positive impact of the pandemic for those with higher education, adjusting for resilience. Compernolle et el. assess differences by cognitive status in changes in mental health from pre- to during the pandemic. Higher 2015 cognitive functioning was associated with greater pandemic loneliness, with decreased pandemic emotional support partly explaining this association. Copeland & Liu investigate the role of 2015 personal networks in receiving instrumental and emotional support during the pandemic, finding that larger and denser pre-pandemic confidant networks each predicted higher odds of receiving various types of support during the pandemic. Wong et al. explore whether and for whom relationship quality changed since the pandemic started. Two-thirds of partnered respondents reported unchanged relationship quality, and Black respondents were more likely to report improved relationship quality. These investigations highlight sub-group differences in older adults’ changes in experiences, behavior, and well-being since the pandemic began.

